# Histologic Analysis of Peri-implant Soft Tissue Around External Hexagon and Morse Taper Implant Connections

**DOI:** 10.4317/jced.63947

**Published:** 2026-03-30

**Authors:** Daniel Sartorelli Marques de Castro, Jefferson David Melo de Matos, Jozely Francisca Mello Lima, Guilherme da Rocha Scalzer Lopes, Mário Alexandre Coelho Sinhoreti, Daher Antonio Queiroz, Carlos do Reis Pereira de Araújo

**Affiliations:** 1 Professor of Prosthodontics, Department of Odontology, Centro Universitário Unichristus UNICHRISTUS, Fortaleza - CE, Brazil; 2 PhD, Department of Biomaterials, Dental Materials and Prosthodontics, São Paulo State University (Unesp), Institute of Science and Technology, São José dos Campos, Sao Paulo, Brazil; 3 Professor of Prosthodontics and Occlusion, Department of Dentistry, Universidade Federal do Ceará UFC, Sobral – CE, Brasil; 4 PhD, Department of Biomaterials, Dental Materials and Prosthodontics, São Paulo State University (Unesp), Institute of Science and Technology, São José dos Campos, Sao Paulo, Brazil; 5 Professor, Department of Restorative Dentistry, Dental Materials Division, Piracicaba Dental School (FOP UNICAMP), Piracicaba, Sao Paulo, Brazil; 6 DDS, MSc, PhD Associate Professor, Department of Restorative Sciences and Public Health Dentistry, Nova Southeastern University College of Dental Medicine, Fort Lauderdale, Florida, USA Adjunct Associate Professor, Department of Restorative Dentistry &amp; Prosthodontics, The University of Texas Health Science Center at Houston (UTHealth) School of Dentistry, Houston, Texas, USA; 7 Adjunct Associate Professor, Department of Restorative Dentistry &amp; Prosthodontics, The University of Texas Health Science Center at Houston (UTHealth) School of Dentistry, Houston, Texas, USA; 8 Professor of Prosthodontics, Department of Dentistry, Faculdade de Odontologia de Bauru, FOB/USP, São Paulo - SP, Brasil

## Abstract

**Background:**

Twenty patients rehabilitated with screw-retained full-arch implant-supported prostheses functioning for at least one year were included.

**Material and Methods:**

This clinical study included 20 patients rehabilitated with screw-retained full-arch implant-supported prostheses in function for at least 1 year. Participants were allocated into two groups according to the implant-abutment connection design: external hexagon (EH; n = 10) and Morse taper (MT; n = 10). One peri-implant soft tissue biopsy specimen was obtained from each patient and processed for hematoxylin-eosin staining. Histological evaluation assessed epithelial hyperplasia, epithelial organization, inflammatory infiltrate intensity, and connective tissue fibrosis. Data normality was assessed using the Shapiro-Wilk test. Intergroup comparisons were performed using the Mann-Whitney U test, with a significance level of = 0.05.

**Results:**

Mean epithelial hyperplasia scores were 2.0 in the EH group and 1.6 in the MT group (P = 0.690). Mean fibrosis scores were 2.2 (EH) and 2.4 (MT) (P = 0.841). Inflammatory infiltrate scores were 0.4 in the EH group and 2.0 in the MT group (P = 0.095). No statistically significant differences were observed between groups for any evaluated parameter. The combined histologic score also showed no significant intergroup difference (P = 0.222).

**Conclusions:**

Within the limitations of this clinical study, peri-implant soft tissue architecture and inflammatory profiles were similar between external hexagon and Morse taper implant-abutment connections in full-arch rehabilitations following at least one year of functional loading.

## Introduction

Implant-supported rehabilitations depend on the long-term stability of peri-implant hard and soft tissues. Beyond accurate surgical placement and proper prosthetic fit, the design of the implant-abutment connection plays a critical role in the biomechanical and biological dynamics at the transmucosal interface. The geometry of the connection influences micromovements, microgap formation, stress distribution, and bacterial infiltration, all of which may impact peri-implant tissue homeostasis (1 - 4). Mechanical studies have shown that the configuration of the implant-abutment connection affects load distribution and interface stability. External hexagon (EH) connections, which feature a butt-joint interface, may allow greater rotational movement under cyclic loading. In contrast, Morse taper (MT) connections provide a conical internal engagement that improves frictional stability (5 - 9). Finite element analyses and in vitro investigations indicate that conical interfaces may decrease crestal stress concentration and reduce microgap formation during functional loading (5 - 7). Microgaps at the implant-abutment interface has been linked to bacterial penetration and the recruitment of inflammatory cells (10 - 16). Sustained microbial colonization may play a role in marginal bone remodeling and alterations of the peri-implant mucosal architecture (10 - 14). While the radiographic and mechanical performance of various connection systems has been extensively studied, clinical evidence regarding their impact on the histologic characteristics of peri-implant soft tissues remains limited. The peri-implant mucosa establishes a biologic seal that differs from the periodontal attachment, comprising a junctional epithelium and an underlying connective tissue zone characterized mainly by parallel-oriented collagen fibers (1 - 4 , 17). The integrity of this barrier may rely on effective local inflammatory regulation as well as mechanical stability at the implant-abutment interface. Consequently, evaluating whether biomechanical differences between connection designs lead to detectable histological changes in peri-implant soft tissues is clinically significant, especially in full-arch rehabilitations exposed to continuous functional loading. Accordingly, the purpose of this study was to evaluate and compare the histologic features of peri-implant soft tissues surrounding EH and MT prosthetic connections. The working hypothesis was that variations in connection design would affect epithelial architecture, inflammatory patterns, and the degree of connective tissue fibrosis.

## Material and Methods

**Figure 1:**
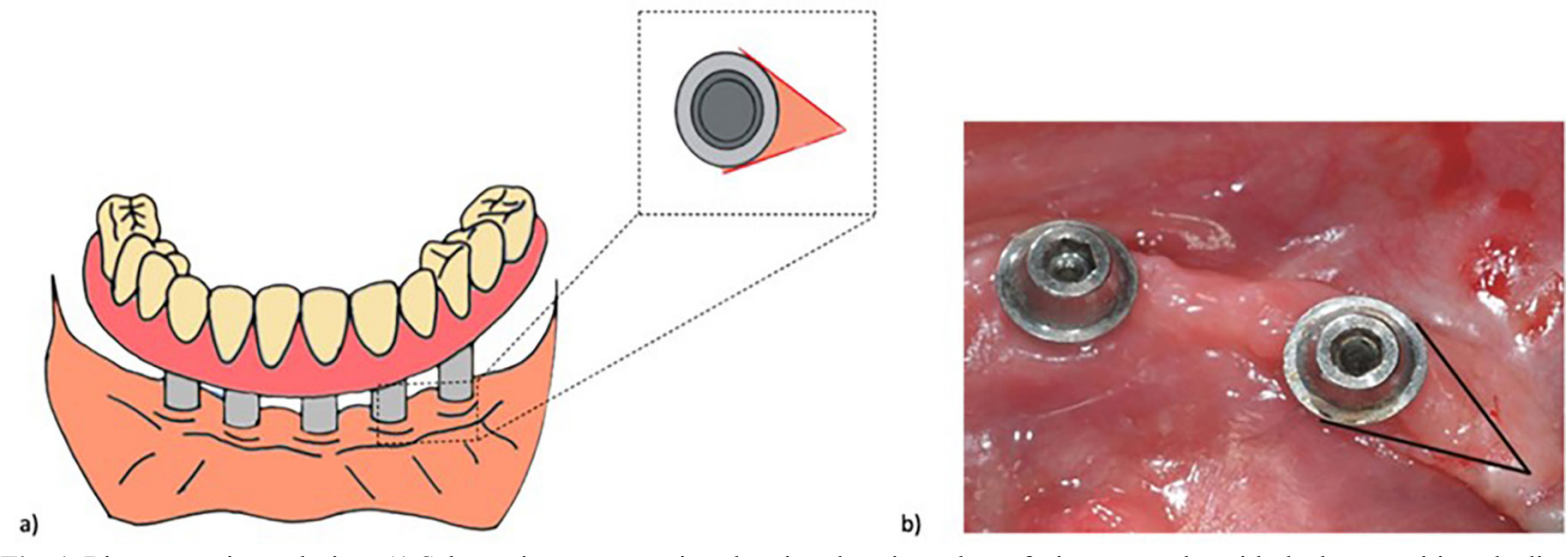
Biopsy specimen design. A) Schematic representation showing the triangular soft tissue sample, with the base positioned adjacent to the prosthetic component. B) Clinical photograph of the excised specimen, measuring approximately 2.0 × 1.5 mm.

1. Study Design and Ethical Considerations This clinical comparative study was conducted in accordance with the Declaration of Helsinki and approved by the Research Ethics Committee of the Bauru School of Dentistry, University of São Paulo (FOB-USP) (protocol No. 12/2006). 2. Patient Selection Twenty patients rehabilitated with screw-retained full-arch implant-supported prostheses functioning for at least one year were recruited from two private dental clinics. Participants were screened according to predefined inclusion and exclusion criteria to ensure peri-implant tissue stability and minimize potential confounding factors that could influence histologic outcomes. Eligible participants were adults (18 years old) presenting full-arch implant-supported prostheses in functional loading for a minimum period of 12 months. Additional inclusion criteria comprised the absence of clinical signs of peri-implantitis, defined as the lack of suppuration, bleeding on probing, or progressive peri-implant bone loss, as well as adequate plaque control, satisfactory oral hygiene, and stable prosthetic function without mechanical complications. Patients were excluded if they presented a history of peri-implantitis or active periodontal disease, systemic conditions known to interfere with wound healing or inflammatory responses (such as uncontrolled diabetes or immunosuppressive disorders), current smoking or a history of heavy smoking, use of medications capable of affecting bone or soft tissue metabolism (including corticosteroids or bisphosphonates), recent antibiotic or anti-inflammatory therapy within the previous three months, poor prosthetic adaptation, or clinical evidence of implant mobility. After eligibility assessment, participants were allocated into two groups according to the implant-abutment connection design present in the rehabilitation: external hexagon (EH; n = 10) and Morse taper (MT; n = 10). The sample size was determined considering both feasibility and ethical aspects related to the invasive nature of peri-implant soft tissue biopsy procedures in clinically healthy implants. Because histologic sampling requires surgical tissue harvesting, the recruitment of large cohorts is ethically restricted in clinical settings. Therefore, a convenience sample of twenty patients was adopted, which is consistent with previously published clinical histologic investigations evaluating peri-implant mucosal tissues. Despite the exploratory nature of the study, this sample size was considered adequate to detect clinically relevant differences in the evaluated histologic parameters between groups. Furthermore, the statistical analysis was performed using nonparametric methods, which are appropriate for small sample sizes and do not require assumptions of normal data distribution. 3. Biopsy Procedure A single peri-implant soft tissue sample was collected from each patient at a transmucosal site directly contacting the prosthetic component. Local anesthesia was administered using 0.5% bupivacaine with epinephrine. A triangular tissue specimen, approximately 2.0 × 1.5 mm in size, was excised with a No. 15C scalpel blade, positioning the base toward the prosthetic interface. The biopsy site was then closed with 5-0 silk sutures, and the specimens were immediately fixed in 10% buffered formalin (Fig. 1).

1

4. Histologic Processing and Evaluation Specimens were embedded in paraffin, cut into 4 m sections, and stained with hematoxylin-eosin. Two oral pathologists, blinded to the group assignments, independently examined the samples. The evaluated parameters included epithelial hyperplasia (using a graded scale), epithelial integrity and organization, presence and severity of inflammatory infiltrate, and the degree of connective tissue fibrosis. 5. Statistical analysis Statistical analyses were performed using SPSS version 29.0 (IBM Corp., Armonk, NY, USA). The Shapiro-Wilk test was applied to assess data distribution. Since normality was not observed, comparisons between groups were conducted using the Mann-Whitney U test. A significance threshold of = 0.05 was adopted.

## Results

**Figure 2:**
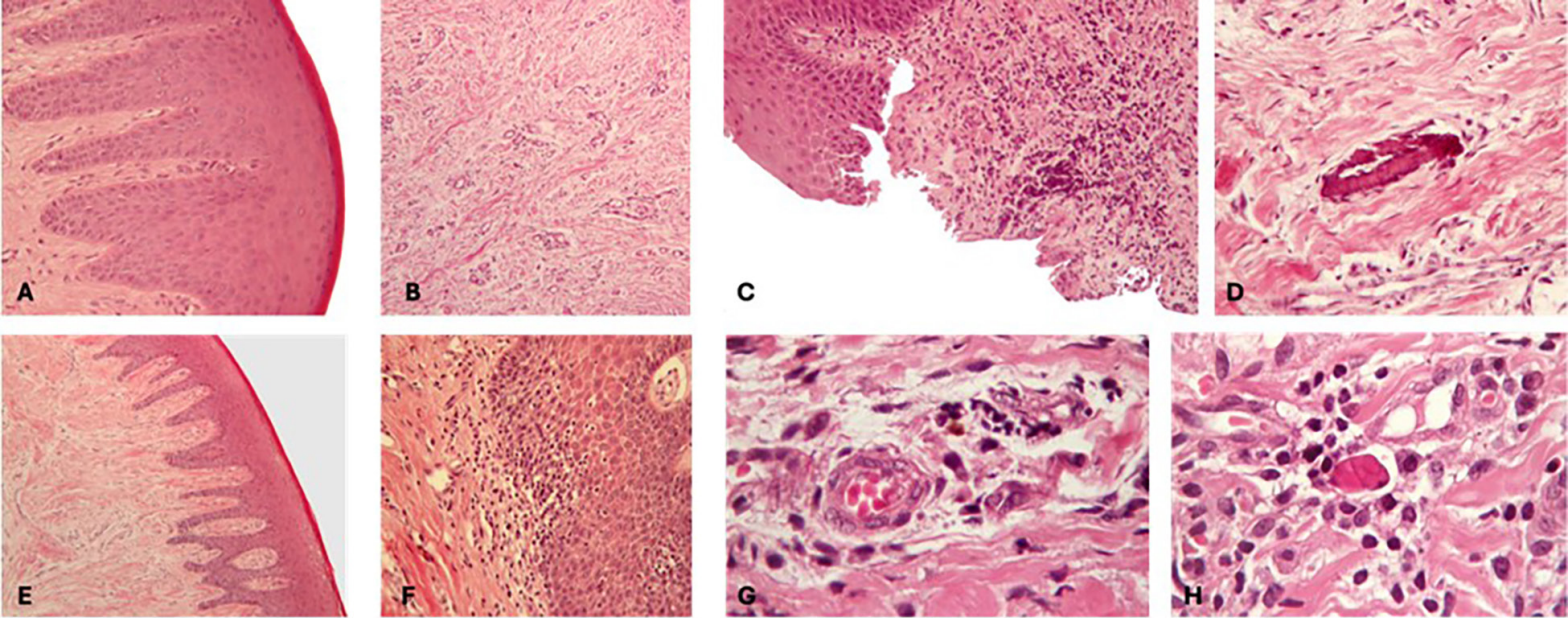
Histologic features of peri-implant soft tissues in external hexagon (EH) and Morse taper (MT) groups. (A) EH group: Moderate epithelial hyperplasia with preserved epithelial organization. (B) EH group: Well-organized connective tissue with dense collagen bundles and absence of inflammatory infiltrate. (C) EH group: Moderate mononuclear inflammatory infiltrate within the lamina propria. (D) EH group: Radicular cementum fragment surrounded by collagen fibers within the connective tissue. (E) MT group: Peri-implant mucosa without epithelial hyperplasia. (F) MT group: Lichenoid inflammatory pattern characterized by a band-like inflammatory infiltrate subjacent to the epithelium. (G) MT group: Deep mononuclear inflammatory infiltrate localized within the lamina propria. (H) MT group: Russell bodies observed within plasma cells in the connective tissue. Hematoxylin–eosin staining; original magnification ×200 (A–G) and ×400 (H).

All biopsy sites healed without complications, and every specimen was deemed suitable for analysis. 1. Epithelial characteristics Epithelial hyperplasia was present in all specimens from the external hexagon (EH) group (n = 10), with a mean score of 2.0. In the Morse taper (MT) group, hyperplasia was observed in 8 out of 10 specimens (mean score 1.6), while 2 specimens showed no epithelial thickening (Fig. 2A-D for EH and Fig. 2E-H for MT).

2

The difference between groups was not statistically significant (P = 0.690) (Table 1).

Table 1

Epithelial integrity was maintained in all EH specimens (Fig. 2 A). In the MT group, 8 specimens showed preserved epithelial organization, whereas 2 exhibited focal epithelial disorganization associated with inflammatory cell infiltration. Additionally, a lichenoid inflammatory pattern was detected in 5 MT specimens but was absent in the EH group (Fig. 2 G). 2. Connective tissue findings All specimens from both groups exhibited well-organized connective tissue with dense collagen bundles and vascular structures (Fig. 2 B). In the EH group, 8 specimens showed no inflammatory infiltrate (Fig. 2C), while 2 displayed moderate inflammatory infiltration (Fig. 2 C). In the MT group, inflammatory infiltrate was present in 6 specimens (60%) and absent in the remaining 4 specimens. The inflammatory infiltrate was primarily composed of mononuclear cells and confined to the lamina propria (Fig. 2 G). Russell bodies were observed in 6 MT specimens (Fig. 2 H). The average fibrosis score was 2.2 in the EH group and 2.4 in the MT group, with no statistically significant difference between groups (P = 0.841) (Table 1). 3. Combined histologic variables When hyperplasia, fibrosis, and inflammatory intensity were evaluated together as a composite score, the mean values were 4.6 for the EH group and 6.0 for the MT group. No statistically significant difference was found between the groups (P = 0.222) (Table 1).

## Discussion

This study assessed the histological features of peri-implant soft tissues surrounding external hexagon (EH) and Morse taper (MT) connections in functionally loaded full-arch rehabilitations. Despite well-established biomechanical differences between the two connection types, no statistically significant differences were found between groups in terms of epithelial hyperplasia, connective tissue fibrosis, inflammatory intensity, or overall combined histologic scores. Biomechanical studies suggest that conical internal connections can offer greater mechanical stability and reduced micromovement compared with external hexagon designs, especially under cyclic loading (5 - 9). Minimizing micromovement may help limit microgap formation and bacterial infiltration at the implant-abutment interface (10 - 16). Nevertheless, under the clinically stable conditions examined in this study, these mechanical differences did not translate into detectable changes in peri-implant mucosal structure. The epithelial hyperplasia observed in both groups aligns with adaptive responses to functional and prosthetic stimuli. Previous studies characterize the peri-implant mucosa as a biologic seal distinct from the periodontal attachment, consisting of a junctional epithelium and a connective tissue layer with primarily parallel-oriented collagen fibers (1 - 4 , 17). The maintained epithelial organization in both groups suggests that implant-abutment connection geometry alone may have minimal impact on epithelial morphology when effective plaque control and prosthetic stability are ensured. Recent investigations have further characterized the histologic and biological behavior of peri-implant soft tissues, highlighting the role of mucosal thickness, collagen fiber orientation, and vascularization in maintaining the peri-implant barrier (23 - 26). Histologic and immunohistochemical studies indicate that peri-implant mucosa exhibits a connective tissue architecture distinct from periodontal tissues, with collagen fibers predominantly oriented parallel to the implant surface and relatively reduced vascular supply (24 , 26). These structural features may influence the local inflammatory response and the stability of the peri-implant epithelial seal (26 , 27). Moreover, current evidence suggests that peri-implant tissue health is multifactorial, being influenced by implant surface characteristics, prosthetic design, and bacterial colonization at the implant-abutment interface, rather than solely by connection geometry (28). Consequently, histologic evaluation remains essential for understanding the biological mechanisms underlying peri-implant tissue adaptation and long-term implant stability (29 , 30). Connective tissue organization and the degree of fibrosis were similar between the groups. While load distribution may differ between EH and MT systems, especially at the crestal level (5 - 9), these variations did not translate into observable differences in soft tissue density or structural arrangement in the present clinical sample. Inflammatory infiltrate, mainly composed of mononuclear cells and confined to the lamina propria, was observed in both groups, with no statistically significant differences. Although microgap-related inflammation has been reported in experimental studies (10 - 16), the current results indicate that the peri-implant inflammatory response in clinically healthy rehabilitations is multifactorial and not solely influenced by the geometry of the implant-abutment connection (19 - 21). From a clinical perspective, these findings suggest that when prosthetic stability and plaque control are properly maintained, both connection types can support similar peri-implant soft tissue organization under functional loading. While in vitro studies emphasize the mechanical advantages of conical interfaces (5 - 9 , 22), these differences may not result in observable histologic changes in clinically stable full-arch rehabilitations. Consequently, selecting an implant-abutment connection should consider prosthetic design, ease of maintenance, and long-term restorative planning, in addition to theoretical biomechanical benefits. This study is limited by its cross-sectional design and the lack of molecular or immunohistochemical analyses, which might detect subtle inflammatory changes not apparent in conventional histology. Future longitudinal studies incorporating biomechanical evaluations, quantitative assessment of inflammatory markers, and crestal bone measurements are needed to better understand the relationship between implant-abutment interface mechanics and peri-implant tissue behavior.

## Conclusions

Within the limitations of this clinical study, no significant histologic differences were observed between external hexagon and Morse taper implant-abutment connections in peri-implant soft tissues after at least one year of functional loading. These results suggest that, in clinically stable full-arch rehabilitations, both connection types provide comparable support for peri-implant mucosal organization. Therefore, the choice of implant-abutment connection should be based on overall restorative planning and clinical considerations rather than anticipated differences in soft tissue histology.

## Figures and Tables

**Table 1:** Comparison of histologic variables between external hexagon and Morse taper groups (Mann–Whitney U test).

Variable	EH (n=10) Mean	MT (n=10) Mean	P value
Epithelial hyperplasia	2.0	1.6	0.690
Fibrosis	2.2	2.4	0.841
Inflammatory intensity	0.4	2.0	0.095
Combined score	4.6	6.0	0.222

Abbreviations: EH, external hexagon; MT, Morse taper. Statistical test: Mann–Whitney U test. Significance level: α=0.05.

## Data Availability

The datasets generated and analyzed during the current study are available from the corresponding author upon reasonable request.
